# (2*E*,5*E*)-2,5-Bis(3-hydroxy-4-methoxybenzylidene) cyclopentanone Exerts Anti-Melanogenesis and Anti-Wrinkle Activities in B16F10 Melanoma and Hs27 Fibroblast Cells

**DOI:** 10.3390/molecules23061415

**Published:** 2018-06-11

**Authors:** Hee Jin Jung, A Kyoung Lee, Yeo Jin Park, Sanggwon Lee, Dongwan Kang, Young Suk Jung, Hae Young Chung, Hyung Ryong Moon

**Affiliations:** 1 Molecular Inflammation Research Center for Aging Intervention (MRCA), Pusan National University, Busan 46241, Korea; hjjung2046@pusan.ac.kr (H.J.J.); lak000@naver.com (A.K.L.); pyjin5526@gmail.com (Y.J.P.); lsk3232@pusan.ac.kr (S.L.); 3607@pusan.ac.kr (D.K.); youngjung@pusan.ac.kr (Y.S.J.); hyjung@pusan.ac.kr (H.Y.C.); 2 College of Pharmacy, Pusan National University, Busan 46241, Korea

**Keywords:** (2*E*,5*E*)-2,5-Bis(3-hydroxy-4-methoxybenzylidene)cyclopentanone (BHCP), tyrosinase inhibitor, anti-melanogenesis, anti-wrinkle

## Abstract

Ultraviolet (UV) radiation exposure is the primary cause of extrinsic skin aging, which results in skin hyperpigmentation and wrinkling. In this study, we investigated the whitening effect of (2*E*,5*E*)-2,5-bis(3-hydroxy-4-methoxybenzylidene)cyclopentanone (BHCP) on B16F10 melanoma and its anti-wrinkle activity on Hs27 fibroblasts cells. BHCP was found to potently inhibit tyrosinase, with 50% inhibition concentration (IC_50_) values of 1.10 µM and 8.18 µM for monophenolase (l-tyrosine) and diphenolase (l-DOPA), and the enzyme kinetics study revealed that BHCP is a competitive-type tyrosinase inhibitor. Furthermore, BHCP significantly inhibited melanin content and cellular tyrosinase activity, and downregulated the levels of microphthalmia-associated transcription factor (MITF), phosphorylated levels of cAMP response element-binding (CREB) protein, and tyrosinase in α-melanocyte stimulating hormone (α-MSH)-induced B16F10 melanoma cells. Moreover, BHCP inhibited the phosphorylation of p65 and expression of matrix metalloproteinases (MMP-1, MMP-9, MMP-12, and MMP-13) in Hs27 fibroblasts stimulated with UV radiation. Therefore, our results demonstrate that BHCP may be a good candidate for the development of therapeutic agents for diseases associated with hyperpigmentation and wrinkling.

## 1. Introduction

Skin aging is a complex and progressive process that leads to functional and aesthetic changes in the skin, with both intrinsic and extrinsic factors being responsible [1]. Extrinsic skin aging is caused by environmental aggressors, such as ultraviolet (UV) radiation, stress, or smoking. However, it is mainly caused by repeated exposure to UV from the sun, which is called photoaging. Skin photoaging is characterized by coarse and deep wrinkles, thickness, roughness, dyspigmentation, and histological changes [2,3,4].

Tyrosinase (EC 1.14.18.1), which is also known as polyphenol oxidase, is one of the multifunctional copper-containing enzymes involved in melanin synthesis and is found widely in nature [5]. Tyrosinase is typically present in a majority of microorganisms, plants, and animals. In plants, tyrosinase acts by oxidizing monophenols into diphenols (monophenolase activity) and is involved in the oxidation of *o*-diphenols into *o*-quinones (diphenolase activity), followed by the oxidation of quinones into dark-brown pigments [6].

Melanogenesis is the transformation of l-tyrosine into 3,4-dihydroxyphenylalanine (l-DOPA), whereby l-DOPA is converted into DOPA quinine [7]. Hence, tyrosinase plays an important role in melanin production in melanocytes, and the inhibition of tyrosinase is an attractive target for the improvement of pigmentation-related disorders and for the development of whitening agents [8,9]. Melanin synthesis is induced by several stimuli, such as UV and chemicals including isobutylmethylxanthine (IBMX) and alpha-melanocyte-stimulating hormone (α-MSH). α-MSH binds to its receptor melanocortin 1 receptor (MC1R), subsequently increasing the cytoplasmic cyclic AMP (cAMP) level. The increased cAMP level activates protein kinase A (PKA), which induces the expression of microphthalmia-associated transcription factor (MITF) via phosphorylation of the cAMP response element-binding protein (CREB). MITF induces the expression of tyrosinase, tyrosinase-related protein (TRP)-1, and TRP-2, which finally results in increased melanin synthesis [10]. MITF is considered a key transcription factor of melanogenesis; therefore, many studies have been performed to control the expression of MITF to inhibit melanogenesis [11].

Wrinkle formation is known to be closely associated with the degradation of the extracellular matrix of the skin, and UV radiation activates nuclear factor-κB (NF-κB), thereby increasing the production of collagen fragmentation and matrix metalloproteinases (MMPs) [2]. MMPs are zinc-dependent endopeptidases that are important in the remodeling of the extracellular matrix structure in skin. Therefore, the excessive degradation of collagen and the matrix by UV-induced MMPs is a characteristic feature of photodamaged skin, and MMPs are used as a major marker of UV-induced photoaging as well as skin inflammation [12].

**Figure 1:**
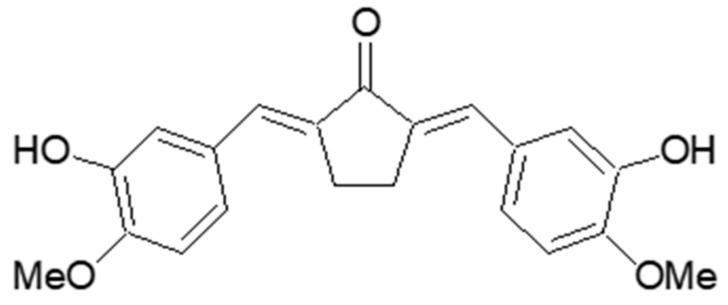
Structure of (2*E*,5*E*)-bis(3-hydroxy-4-methoxybenzylidene)cyclopentanone (BHCP).

The curcumin-like diarylpentanoid skeleton derivatives have been reported to exhibit a wide range of bioactivities, including antioxidant, anticancer, anti-inflammation, anti-melanogenesis, and anti-tyrosinase [13,14,15,16,17,18] activities. Especially, Leow et al. [19] reported in 2014 that dibenzylidene-cyclopentanone scaffolds, including (2*E*,5*E*)-2,5-bis(3-hydroxy-4-methoxybenzylidene)cyclopentanone (BHCP) (Figure 1), might contribute to the protective effects on human osteosarcoma via regulation of the Wnt/β-catenin signaling pathway. However, the anti-melanogenesis and anti-wrinkle effects of BHCP remain to be discovered, and the molecular mechanisms underlying its activity have not yet been clearly established. 

In the present study, we investigated the anti-melanogenesis and anti-wrinkle potential of BHCP, and sought to identify the mechanism involved, especially with respect to melanin content and cellular tyrosinase activity, which were explored using tyrosinase inhibition assay and analysis of enzyme kinetics. Moreover, we demonstrated that BHCP exerts an inhibitory effect on melanogenesis and wrinkles that is associated with the downregulation of CREB/MITF/tyrosinase in α-MSH-induced B16F10 mouse melanoma cells and inhibition of phosphorylation of p65 and MMP expression in UV-induced Hs27 human fibroblasts.

## 2. Results and Discussion

### 2.1. Synthesis of (2E,5E)-2,5-Bis(3-hydroxy-4-methoxybenzylidene)cyclopentanone (BHCP)

A solution of 3-hydroxy-4-methoxybenzaldehyde (100 mg, 0.66 mmol, isovanillin) and cyclopentanone (0.03 mL, 0.33 mmol) in 1 N HCl-acetic acid solution (0.02 mL) was stirred at 25 °C for 2 h. After standing for 1 day, the reaction mixture was treated with cold water in the presence of a small amount of methanol, filtered, and washed with cold water to produce BHCP (65.9 mg) in 56.9% yield. BHCP was identified by spectroscopic methods, including ^1^H and ^13^C-NMR, as well as by comparison with published spectral data and Thin Layer Chromatography (TLC) analysis [19]. The structure is shown in Figure 1.

BHCP: yellow amorphous powder (CHCl_3_); ^1^H-NMR (500 MHz, DMSO-*d*_6_) δ: 9.25 (s, 2H, 2 × OH), 7.28 (s, 2H, 2 × vinylic H), 7.14 (d, 2H, *J* = 2.0 Hz, 2 × 2-H), 7.11 (dd, 2H, *J* = 8.5, 2.0 Hz, 2 × 6-H), 7.01 (d, 2H, *J* = 8.5 Hz, 2 × 5-H), 3.81 (s, 6H, 2 × OMe), 3.01 (s, 4H, 2 × CH_2_); ^13^C-NMR (100 MHz, DMSO-*d*_6_) δ: 195.5, 149.9, 147.2, 136.0, 133.1, 129.1, 124.3, 117.5, 112.8, 56.3, 26.6; ESI-MS: *m*/*z* 351 (M − H)^−^.

### 2.2. Inhibitory Effect of BHCP on Mushroom Tyrosinase Activity

As shown in Table 1, BHCP inhibited tyrosinase with IC_50_ values of 1.10 ± 0.12 µM and 8.18 ± 0.44 µM, whereas kojic acid (positive control) had IC_50_ values of 18.68 ± 1.40 µM and 33.89 ± 1.16 µM for monophenolase and diphenolase, respectively. In our previous study of synthetic potential inhibitors of tyrosinase, we found the mechanism through molecular modeling studies by which the 3-hydroxy and 4-methoxy groups of benzylidene had great binding tendencies towards the tyrosinase active site [20]. From this study result, it was shown that its functional groups (3-hydroxy and 4-methoxy groups) were associated with a significant increase in the tyrosinase inhibitory activity.

**Figure 2:**
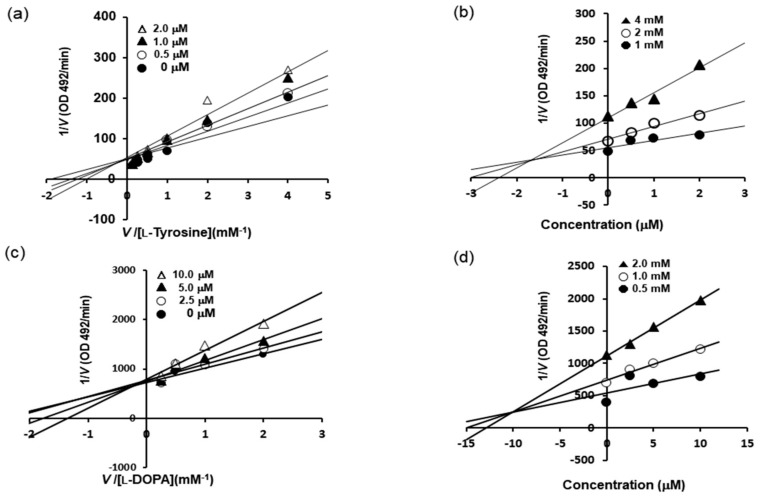
Lineweaver–Burk (**a**,**c**) and Dixon (**b**,**d**) plots for tyrosinase enzyme inhibition by BHCP. Tyrosinase inhibition was analyzed in the presence of different sample concentrations as follows: 0 μM (filled circles), 0.5 μM (open circles), 1 μM (filled triangles), and 2 μM (open triangles) (**a**); and effect in the presence of different concentrations of substrate (l-tyrosine): 1 mM (filled circles), 2 mM (open circles), and 4 mM (filled triangles) (**b**); Tyrosinase inhibition was analyzed in the presence of different sample concentrations as follows: 0 μM (filled circles), 2.5 μM (open circles), 5 μM (filled triangles), and 10 μM (open triangles) (**c**); and effect in the presence of different concentrations of substrate (l-DOPA): 0.5 mM (filled circles), 1.0 mM (open circles), and 2.0 mM (filled triangles) (**d**).

Furthermore, the mechanism responsible for tyrosinase inhibition by BHCP was investigated by enzyme kinetic analysis in the present study (Table 1 and Figure 2). Lineweaver–Burk plots were drawn using the data obtained from the kinetic studies, and the inhibition constant (*K*_i_) was obtained from the Dixon plots. The Lineweaver–Burk double reciprocal plots indicated competitive-type inhibition. As shown in Figure 2a–c, BHCP acted as a competitive inhibitor of both l-tyrosine and l-DOPA. Moreover, intercepts on the *x*-axis of Dixon plots are commonly used to determine the types of enzyme inhibition constants (*K*_i_) for an enzyme–inhibitor complex [21,22], where the value of the *x*-axis indicates the value of −*K*_i_. As depicted in Figure 2b–d, the *K*_i_ values of BHCP were 1.7 µM and 10.5 µM as substrates for l-tyrosine and l-DOPA, respectively. As the *K*_i_ value represents the concentration required to form an enzyme–inhibitor complex, a lower *K*_i_ value suggests more effective inhibition against tyrosinase. 

### 2.3. Effects of BHCP on the Cell Viability of B16F10 Melanoma and Hs27 Fibroblast Cells

**Figure 3:**
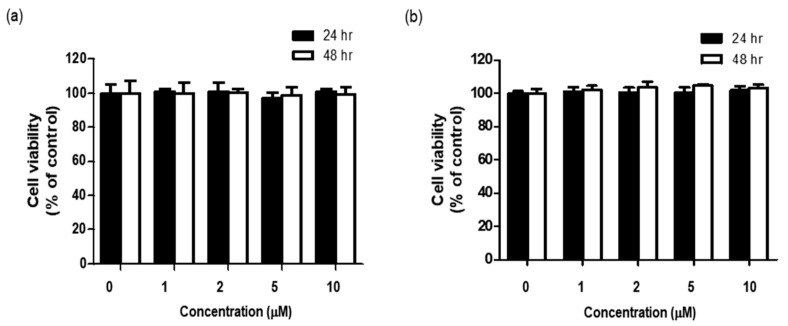
Cell viability of BHCP on B16F10 melanoma (**a**) and Hs27 fibroblast cells (**b**). Cells were treated with various concentrations (0–10 μM) of BHCP for 24 h and 48 h, respectively, and cell viability was measured by EZ-Cytox assay.

Before determining whether BHCP exerted any anti-melanogenesis and anti-wrinkle activities, we examined the cytotoxicity of BHCP to B16F10 cells and Hs27 cells by treatment with different concentrations of BHCP for different time intervals, and cell viability was measured with the EZ-Cytox assay. As shown in Figure 3a,b, up to 10 µM of BHCP for 48 h did not reduce the survival of either the B16F10 cells or Hs27 cells. Subsequently, further in vitro studies on the anti-melanogenesis and anti-wrinkle activities of BHCP were conducted with 1, 5, and 10 µM.

### 2.4. Inhibition of BHCP against Melanin Content and Cellular Tyrosinase Activity in B16F10 Melanoma Cells

**Figure 4:**
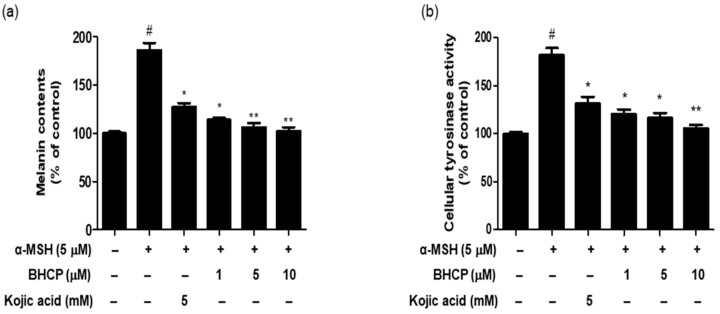
Inhibition of melanin contents (**a**) and cellular tyrosinase activity (**b**) of BHCP on B16F10 melanoma cells. Cells were preincubated with various concentrations (1 μM, 5 μM, and 10 μM) of BHCP or kojic acid (5 mM) for 24 h and then stimulated with α-MSH (5 μM) for 48 h. Melanin content of the pellet was evaluated at 405 nm (**a**); intracellular tyrosinase activity was determined with l-DOPA as the substrate, and the absorbance of l-DOPA chrome was read at 492 nm (**b**). Data are expressed as mean ± S.E.M of three independent experiments. One-way ANOVA was used to determine the significances of differences: ^#^ *p* < 0.05 compared to the control group without α-MSH treatment. ** *p* < 0.01 and * *p* < 0.05 compared to the α-MSH-treated control group.

To determine whether BHCP exerts inhibitory potential on the melanin content of α-MSH-induced B16F10 cells, cells were pretreated with the indicated different concentrations (1, 5, and 10 µM) of BHCP or kojic acid (5 mM) for 24 h and then stimulated with α-MSH for 48 h. As shown in Figure 4a, the melanin content in the cells treated with BHCP in the presence of α-MSH decreased in a concentration-dependent manner, showing 113% at 1 µM, 106% at 5 µM, and 102% at 10 µM, compared to the control group treated with α-MSH only (186%). Interestingly, BHCP (1 µM) inhibited melanin content more strongly than kojic acid (5 mM). Furthermore, a cellular tyrosinase activity assay was performed to measure the inhibitory effect of BHCP on B16F10 cells. As shown in Figure 4b, BHCP decreased in a concentration-dependent manner with the tyrosinase activity by 120% at 1 µM, 116% at 5 µM, and 105% at 10 µM, compared to the control group treated with α-MSH only (181%). The inhibitory effect of BHCP was much more potent than that of kojic acid; the inhibition of BHCP at 1 µM was superior to that of kojic acid at 5 mM (131%). These results suggest that BHCP has a whitening effect by inhibiting melanin biosynthesis and intracellular tyrosinase synthesis in B16F10 melanocytes. 

### 2.5. Effects of BHCP on the Expression of MITF/Tyrosinase and Phosphorylated CREB in B16F10 Cells

**Figure 5:**
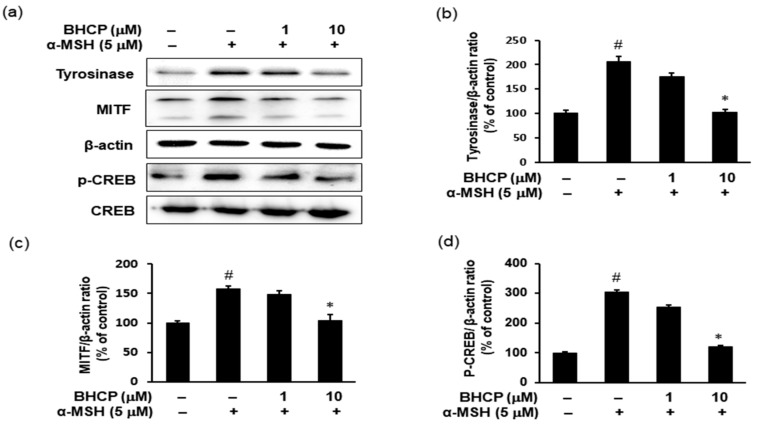
Effects of BHCP on the expression levels of phosphorylation of CREB, MITF, and tyrosinase in α-MSH-stimulated B16F10 melanoma cells. Cells were preincubated with or without 1 and 10 μM BHCP for 24 h and were stimulated with α-MSH (5 μM) for an additional 48 h. Harvested cells were lysed and then examined for the expression levels of p-CREB, CREB, MITF, and tyrosinase by Western blotting (**a**); The protein levels of tyrosinase (**b**); MITF (**c**); and p-CREB (**d**) were quantitated with CS analyzer software. Tyrosinase, MITF, and p-CREB protein levels were normalized by β-actin. Data are expressed as mean ± S.E.M. of three independent experiments. ^#^ *p* < 0.05 versus untreated control; * *p* < 0.05 versus α-MSH stimulation.

MITF, a specific transcription factor, plays a pivotal role in effectively activating the melanogenic genes, including tyrosinase, catalyzing the rate-limiting step in melanin biosynthesis: TRP-1, and TRP-2. The expression of MITF could be increased by the phosphorylation of CREB [23]. CREB is an important MITF promoter [24,25], and the phosphorylation of CREB in melanocytes increases MITF expression by binding to the CREB (c-AMP response element-binding protein) in melanocytes [26]. To elucidate the molecular pathways responsible for the anti-melanogenic effect of BHCP on B16F10 cells, we examined the protein levels of key molecules, including CREB and MITF, that play important roles in melanogenesis by Western blot analysis. The cells were treated with BHCP or kojic acid and then stimulated by α-MSH for 48 h. The time interval for the measurements followed the methodology described in previous studies [27]. As shown in Figure 5, tyrosinase and MITF levels increased with α-MSH, but BHCP decreased these protein levels. Furthermore, phosphorylation of CREB was significantly suppressed by BHCP. The regulation of α-MSH-induced CREB phosphorylation is known to be potentially important in regulating pigmentation [28]. These results indicate that the anti-melanogenic effects of BHCP result from the downregulation of MITF and tyrosinase via the downregulation of phosphorylated CREB. The present study suggests that the elucidation of the inhibition mechanism of BHCP on tyrosinase and melanogenesis is crucial and must be further investigated in the future. Although the B16F10 melanoma cell line is generally more suitable for investigating signaling mechanisms in vitro, the B16F10 cell line is of a rodent melanoma. Therefore, further studies on human melanocytes will be necessary to confirm the findings. 

### 2.6. Effect of BHCP on UV-Induced NF-κB p-p65 Activation in Hs27 Cells

**Figure 6:**
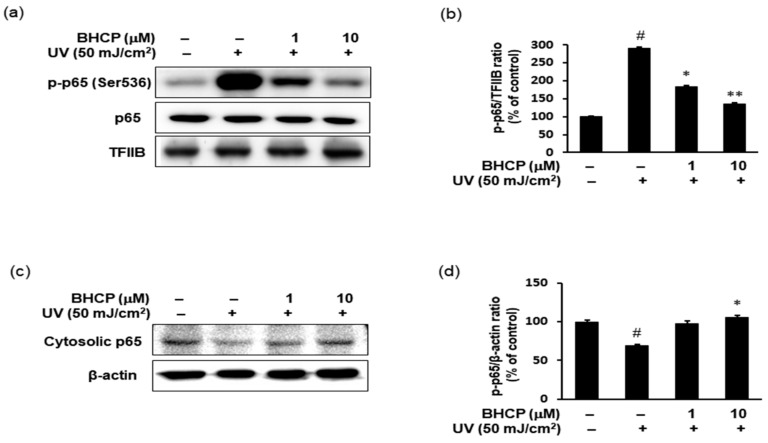
Effects of BHCP on the expression levels of p-p65 (Ser536) in UV-induced Hs27 human fibroblast cells. Cells were preincubated with or without 1 and 10 μM BHCP for 24 h and were stimulated with UV (50 mJ/cm^2^) for an additional 24 h. Nuclear protein levels of p-p65 (Ser536) were determined by Western blotting (**a**); After normalization to TFIIB, relative ratios were quantitated with CS analyzer software (**b**); Cytosol protein levels of p65 was determined by Western blotting (**c**); After normalization to β-actin, ratios were quantitated with CS analyzer software (**d**). Data are expressed as mean ± S.E.M. of three independent experiments. ^#^ *p* < 0.05 versus untreated control; * *p* < 0.05 and ** *p* < 0.01 versus α-MSH stimulation.

We next investigated the effect of BHCP on UV-induced expression of inflammatory mediators using Hs27 fibroblasts. It has been reported previously that the phosphorylation of p65 (Ser536) is essential for its capacity to transactivate genes [29]. Therefore, the protein levels of p-p65 (Ser536) and p65 were examined in the nucleus fraction by Western blotting. As indicated in Figure 6a,b, in Hs27 cells, UV increased the protein levels of p-p65 (Ser536) in the nucleus, whereas treatment of BHCP decreased the nucleus p-p65 (Ser536) protein levels. Correspondingly, the total amount of p65 was decreased in the cytoplasm by UV and restored by BHCP (Figure 6c,d). Although the protein expression levels of p65 were decreased in the cytoplasm and increased in the nucleus after UV induction, pretreatment with BHCP reversed these trends in a dose-dependent manner. Thus, these results suggest that the inhibition of p-p65 by BHCP might contribute to the protective effects on skin pigmentation and collagen destruction against UV.

### 2.7. Effect of BHCP on the Expression of MMPs in Hs27 Cells

**Figure 7:**
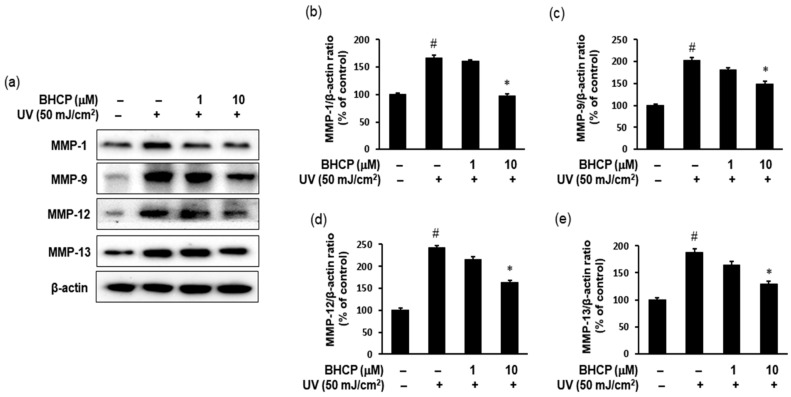
Effects of BHCP on the expression levels of MMP-1, MMP-9, MMP-12, and MMP-13 in UV-induced Hs27 human fibroblast cells. Cells were preincubated with or without 1 and 10 μM BHCP for 24 h and were stimulated with UV (50 mJ/cm^2^) for an additional 24 h. Harvested cells were lysed and then examined for the expression levels of MMP-1, MMP-9, MMP-12, and MMP-13 by Western blotting (**a**); The protein levels of MMP-1 (**b**); MMP-9 (**c**); MMP-12 (**d**); and MMP-13 (**e**) were quantitated with CS analyzer software. MMP-1, MMP-9, MMP-12, and MMP-13 protein levels were normalized by β-actin. Data are expressed as mean ± S.E.M. of three independent experiments. ^#^ *p* < 0.05 versus untreated control; * *p* < 0.05 versus UV stimulation.

MMPs play a vital role in skin aging. UV irradiation alters the connective tissues of the skin by upregulating the expression of MMPs [30,31]. MMP-1 is a collagen-decomposing enzyme that accelerates the breakdown of collagen synthesized from type I procollagen. MMP-9 is a gelatin-decomposing enzyme that breaks down collagen fibers cut by MMP-1, increasing wrinkle production and elasticity loss. To examine the anti-wrinkle effect of BHCP against UV induction, we determined the levels of MMP-1 and MMP-9 protein by Western blotting. Furthermore, UV-induced cells showed significantly elevated level of MMP-13, which initiates the degradation of type I and III collagen instead of MMP-1 [32]. We investigated the increase of MMPs (MMP1, MMP9, MMP12, and MMP13) after treatment of UV-induced Hs27 cells with BHCP at 1 and 10 µM. As shown in Figure 7, levels of MMP-1, MMP-9, MMP-12, and MMP-13 expression increased after UV induction; however, BHCP treatment decreased expression in a dose-dependent manner. Our results suggest that BHCP might contribute to the prevention of wrinkle formation by reducing the abnormal production of MMPs induced by UV exposure. These results imply that BHCP inhibits MMP expression in Hs27 fibroblasts to prevent collagen decomposition, and thus wrinkle production. Therefore, BHCP exhibits the potential for use as a preventive and treatment drug of skin-related disease.

## 3. Material and Methods

### 3.1. Chemicals and Instrumentation

Mushroom tyrosinase (EC 1.14.18.1), α-MSH, l-tyrosine, 3,4-dihydroxyphenylalanine (l-DOPA), dimethyl sulfoxide (DMSO), and kojic acid were purchased from Sigma-Aldrich (St. Louis, MO, USA). Dulbecco’s modified Eagle’s medium (DMEM), fetal bovine serum, streptomycin, and amphotericin were purchased from Gibco Life Technologies Inc. (Carlsbad, CA, USA). Antibodies against MITF, CREB, p-CREB, p-p65 (Ser536), p65, tyrosinase, MMP-1, MMP-9, MMP-12, MMP-13, TFIIB, and β-actin were purchased from Santa Cruz Biotechnology (Santa Cruz, CA, USA). Polyvinylidene difluoride (PVDF) membranes were obtained from the Millipore Corporation (Bedford, MA, USA). Sterile plastic ware for tissue cultures was purchased from SPL Labware (Seoul, Korea). The UV light source was provided by the Crosslinker 800 series (UVP, CA, USA) 6 lamp unit (8 watts/lamp). Thin-layer chromatography and silica gel 60 (mesh 230–400) were performed on silica gel F_254_-precoated plates from Merck Millipore (Darmstadt, Germany). NMR spectra were recorded using a Varian Unity INOVA 400 (400 MHz for ^1^H, 100 MHz for ^13^C) and Varian Unity AS 500 (500 MHz for ^1^H) instruments. Chemical shift values (δ) are reported with reference to the respective residual solvent or deuterated peaks (δ_H_ 2.50 and δ_C_ 39.51 for DMSO). Low-resolution mass spectrometry data were obtained with an Expression CMS mass spectrometer (Advion, Ithaca, NY, USA).

### 3.2. Mushroom Tyrosinase Inhibition Assay 

Mushroom tyrosinase inhibitory activity was determined using both l-tyrosine and l-DOPA as substrates, based on the procedure described by Jung et al. [23]. Briefly, 190 µL of tyrosinase enzyme (1000 U diluted with mushroom tyrosinase buffer, including 1 mM l-tyrosine and l-DOPA solution) was added, in the presence or absence of compounds (final concentration ranging from 1 to 20 µM, dissolved in 100% DMSO), to each well of a 96-well plate, to provide a final volume of 200 μL. The plate was incubated at 37 °C for 30 min. Tyrosinase activity was quantified by measuring the absorbance at 492 nm using a microplate reader (TECAN, Salzburg, Austria) and the percentage inhibition (%) was obtained from the following equation:

% inhibition = (Ac − As)/Ac × 100,(1)

where Ac is the absorbance of the control and As is the absorbance of the sample. The IC_50_ values were calculated from the log-linear curves and their equations. Average results for three determinations are shown. Kojic acid was used as a positive control.

### 3.3. Kinetic Analysis of Tyrosinase Inhibition

To determine the kinetic mechanisms, two kinetic methods (Lineweaver–Burk and Dixon plots) were complementarily used [21,22,33]. For the Lineweaver–Burk double reciprocal plots (a plot of 1/enzyme velocity (*1/V*) versus 1/substrate concentration (1/[S])), the inhibition type was determined using various concentrations of l-tyrosine (1, 2, and 4 mM) and l-DOPA (0.5, 1, and 2 mM) as substrates in the presence of different concentrations of BHCP. The concentrations of BHCP were as follows: 0, 0.5, 1.0, and 2.0 µM for l-tyrosine; and 0, 2.5, 5, and 10 µM for l-DOPA. The Dixon plot is a graphical method (plot of 1/enzyme velocity (*1/V*) against inhibitor concentration (I)) for the determination of the type of enzyme inhibition and was used to determine the dissociation constant or *K*_i_ for the enzyme–inhibitor complex. Dixon plots (single reciprocal plots) of the inhibition were obtained in the presence of l-tyrosine substrate at 1, 2, and 4 mM and 0, 0.5, 1.0, and 2.0 µM for BHCP; and l-DOPA substrate at 0.5, 1.0, and 2.0 mM and 0, 2.5, 5.0, and 10.0 µM for BHCP.

### 3.4. Cell Lines and Cell Culture 

Murine B16F10 melanoma cells were obtained from the Korean Cell Line Bank. The human skin fibroblast cell line Hs27 was purchased from the American Type Culture Collection (ATCC, Manassas, VA, USA). These cells were maintained in DMEM supplemented with 10% fetal bovine serum, 100 U/mL penicillin, and 100 mg/mL streptomycin in a humidified 5% CO_2_ incubator at 37 °C. Dermal fibroblasts on a 100 mm dish were treated with BHCP and exposed to 50 mJ/cm^2^ UV in serum-free DMEM (UV light source, UVP). The Hs27 cells were cultured to 70–80% confluence in a 100 mm diameter plate and were used between passage numbers 5 and 15.

### 3.5. Cell Viability Assay

The viability of cells was assessed using the EZ-Cytox kit assay. In brief, B16F10 cells and Hs27 fibroblasts were seeded into a 96-well plate at a density of 1 × 10^4^ cells/well and incubated at 37 °C for 24 h. The cells were fed fresh, serum-free DMEM that contained different concentrations (0, 1, 2, 5, and 10 µM) of BHCP, and incubated for 24 and 48 h. Subsequently, 10 μL of EZ-Cytox solution was loaded into each well and the cells were incubated for 2–4 h. The absorbance measurement of cells in the absence of any treatment was regarded as 100% cell survival. Each treatment was performed in triplicate and each experiment was repeated three times.

### 3.6. Determination of Melanin Contents Assay

The effect of BHCP on α-MSH-induced melanogenesis in B16F10 cells was based on a previously used method with slight modifications [34]. Briefly, B16F10 cells (5 × 10^4^ cells/well) in 6-well plates were allowed to grow to 70–80% confluence. The cells were then treated with different concentrations of BHCP (1, 5, and 10 µM) or kojic acid (5 mM) for 24 h, and then stimulated with α-MSH (5 µM) for 48 h. After treatment, the cells were washed twice with ice-cold PBS, dissolved in 90 µL 1 M NaOH solution including DMSO (5%) at 60 °C for 1 h, and the absorbance was measured at 405 nm with a microplate spectrophotometer (TECAN, Salzburg, Austria). To measure the amount of melanin in the experiment, the rate of inhibition in the treatment groups were calculated from the absorbance of the known concentrations of synthetic melanin, which were corrected to the total amount of protein that was present in the supernatant of the cell lysates. The absorbance of untreated cells was measured in triplicate. 

### 3.7. Cellular Tyrosinase Activity Assay

Cellular tyrosinase activity assay was performed by measuring the rate of oxidation of l-DOPA [35]. B16F10 cells at a density of 5 × 10^4^/cells were placed in 6-well dishes and incubated overnight. The cells were then treated with various concentrations of BHCP (1, 5, and 10 µM) or kojic acid (5 mM) for 24 h, and then stimulated with α-MSH (5 µM) for 48 h. The cells were washed with PBS and lysed in a solution containing 100 µL of 50 mM phosphate buffer (pH 6.5), 0.1 mM phenylmethylsulfonylfluoride (PMSF), and 1% Triton X-100. Then, the cells were placed in a deep freezer machine (−80 °C) for 30 min. After defrosting the cells, the cellular extracts were purified by centrifugation at 12,000 rpm for 30 min at 4 °C. A total of 80 µL the supernatant and 20 µL of l-DOPA (2 mg/mL) were added to a 96-well plate, and the absorbance at a wavelength of 492 nm was measured every 10 min during 1 h at 37 °C with an ELISA plate reader (TECAN, Salzburg, Austria).

### 3.8. Preparation of Cytosolic and Nuclear Extracts of Hs27 Cells

The Hs27 cells were washed with ice-cold PBS and harvested. A buffer containing 10 mM Tris (pH 8.0), 1.5 mM MgCl_2_, 1 mM DTT, 0.1% NP-40, and protease inhibitors was used for the extraction of cytosolic fractions by centrifugation at 12,000 rpm at 4 °C for 15 min, and nuclear fractions were extracted from pellets using a buffer containing 10 mM Tris, 50 mM KCl, 100 mM NaCl, and protease inhibitors, incubated on ice for 30 min, and then centrifuged at 13,000× *g* for 30 min at 4 °C to obtain nuclear fractions. 

### 3.9. Western Blotting

Lysate samples were boiled for 10 min in gel-loading buffer (125 mM Tris-HCl, 4% sodium dodecyl sulfate (SDS), 10% 2-mercaptoethanol, and 0.2% bromophenol blue; pH 6.8) at a volume ratio of 1:1. The total protein equivalents for each sample were separated by SDS-polyacrylamide gel electrophoresis (PAGE) using acrylamide gels as based on the procedure described by Laemmli [36] and transferred to PVDF membranes at 80 V for 2 h using the wet-transfer system. The membranes were immediately placed into a blocking buffer (5% nonfat milk) in 10 mM Tris, pH 7.5, 100 mM NaCl, and 0.1% Tween-20. The blots were blocked to prevent nonspecific binding at 25 °C for 2 h. Subsequently, the membranes were incubated with a specific primary antibody at 4 °C overnight, followed by incubation with a horseradish peroxidase-conjugated secondary antibody at 25 °C for 1 h. Antibody labeling was detected by enhanced chemiluminescence in accordance with the manufacturer’s instructions. Protein quantitation was performed using the Davinch-Chemi TM. Chemiluminescence Imaging System CAS-400SM (Core Bio, Seoul, Korea). Prestained protein markers were used for the determination of molecular weight.

### 3.10. Statistical Analysis

All data are presented as mean ± S.E.M. The data were analyzed by one-way analysis of variance (ANOVA) for the differences between treatments followed by the Bonferroni post-hoc test. A value of *p* < 0.05 was considered statistically significant. 

## 4. Conclusions

In summary, the findings of the present study demonstrated that BHCP has a skin-whitening effect via inhibition of tyrosinase, which is a key enzyme for melanin biosynthesis in α-MSH-induced B16F10 melanocytes. Furthermore, BHCP decreased the expression of MMP protein levels in UV-induced fibroblasts, which is expected to have an anti-wrinkle effect. Further research is required to confirm the whitening and anti-wrinkle effects of BHCP via animal and clinical studies. Finally, BHCP was identified to have both whitening and anti-wrinkle effects, indicating its potential for the development of therapeutic agents for diseases associated with hyperpigmentation and wrinkling. 

## Figures and Tables

**Table 1:** Tyrosinase inhibitory activity and enzyme kinetic analysis of BHCP.

	l-Tyrosine		l-DOPA	
	IC_50_ (µM) ^a^	Inhibition Type ^b^ (*K*_i_, µM) ^c^	IC_50_ (µM) ^a^	Inhibition Type ^b^ (*K*_i_, µM) ^c^
BHCP	1.10 ± 0.12	Competitive (1.75)	8.18 ± 0.44	Competitive (10.5)
Kojic acid ^d^	18.68 ± 1.40	Not tested	33.89 ± 1.16	Not tested

^a^ The 50% inhibitory concentration (IC_50_) were calculated from a log dose inhibition curve and expressed as mean ± S.E.M. of triplicate experiments. ^b^ Determined by the Lineweaver–Burk plot. ^c^ Determined by the Dixon plot. ^d^ Kojic acid was used as a positive control.
